# Influence of southern pine beetle on fungal communities of wood and bark decomposition of coarse woody debris in the New Jersey pine barrens

**DOI:** 10.48130/FR-2021-0017

**Published:** 2021-10-25

**Authors:** John Dighton, Emily Walsh, Glen Groben, Ning Zhang

**Affiliations:** 1 Rutgers, The State University of New Jersey, Department of Ecology, Evolution and Natural Resources; Department of Biology, Camden and Pinelands Field Station, 501 Four Mile Road New Lisbon, NJ 08064, USA; 2 Rutgers, The State University of New Jersey, Department of Plant Biology, 59 Dudley Road, New Brunswick, NJ 08901, USA

**Keywords:** Bark, Pine, Beetle, Next-generation sequencing, Pine barrens, Fungal community

## Abstract

Dead coarse woody debris (fungal food resources) on the forest floor is an ignition source for forest fires. The rate of decomposition of the debris is largely influenced by fungi, determining its residence time on the forest floor. We asked if southern pine bark beetle (*Dendroctonus frontalis*) attack of pitch pine (*Pinus rigida*) alters the decomposition and fungal community of dead woody resources. Wood and bark from beetle infested and non-beetle infested resources were decomposed in litter bags on the forest floor. Decomposition was measured as mass loss and the fungal community by next-generation (PCR and Illumina metabarcoding) sequencing. Bark decomposed slower than wood and resources colonized by beetles decomposed faster than resources with no beetles. The initial differences in fungal communities colonizing the resources continued throughout the 42 months of decomposition. Fungal diversity was higher in wood than bark in initial decay stages, but significantly lower in wood than bark at the end of the 42 month incubation. In contrast, there were no significant differences in fungal communities between beetle infested and uninfested resources. The rate of decomposition of woody resources on the forest floor has great implications for the longevity of fuel sources for forest fires, however, our results indicate that beetle attacked wood poses no greater fire risk than other dead coarse woody debris regarding the residence time.

## INTRODUCTION

The southern pine beetle (SPB), *Dendroctonus frontalis* (Zimm. Colepotera: Curculionidae) is increasing its ecological range by moving northwards as minimal winter temperatures increase^[1, 2]^. Over the past decade, SPB has been increasing in abundance in south and central New Jersey (NJ), where it causes tree death. In 2010, New Jersey Department of Environmental Protection aerial detection surveys documented over 14,000 acres of mortality in that year alone. With its northward movement, SPB transitions from its usual hosts — loblolly (*Pinus taeda*) and shortleaf pine (*Pinus echinata*) — to pitch pine (*Pinus rigida*), the dominant conifer species in the New Jersey pine barrens. Although SPB is a native insect of the USA, its movement into New Jersey is not unlike that of an invader. With potentially "naïve" host trees, limited scientific knowledge, and management policies and practices unused to dealing with large outbreaks^[3]^, this beetle has the potential to cause significant damage to large tracts of the NJ pine barrens^[2]^.

Both the beetle induced death of trees and the management strategy of tree felling results in a greater than normal accumulation of both standing dead and felled timber of a variety of size classes that add to the potential fuel load of the forest floor, increasing the chance of ignition and the severity of wildfires, once ignited. The coarse and fine woody debris decompose slowly and their rate of decomposition is largely dictated by the community of fungi that affect the decomposition^[4]^. The beetle kills entire trees within patches of one to many acres. Adoption of a 'cut and leave' suppression method, greatly increases the volume coarse woody debris on the forest floor in this fire prone ecosystem. The rate of decomposition of coarse woody debris becomes an important factor in regulating the occurrence of wildfire in this ecosystem^[5]^, which varies according the ratio of rotted and sound wood^[6]^. In addition to just producing fuel for fire initiation, bark beetle attack has also been shown to affect potential fire behavior with reduced foliage water content and increased and flammable sugar, crude fat, lipids and terpenes^[7]^, leaving behind higher levels of charcoal in partially burned snags^[8]^.

Trees killed by SPB might tend to have reduced pitch (resin) content, because, depending on the intensity of beetle attacks and the physiological condition of trees, beetle attacks can exceed tree capacity for synthesis of new resin or accumulate excess resin despite being killed by beetles^[9]^. In this case, wood rotting fungi are likely to invade more rapidly and, hence increase the rate of woody residue decomposition (see interactions of defense chemistry and fungal invasion interactions. See review of Six & Wingfield)^[10]^.

It is known that these beetles introduce a number of ascomycete and basidiomycete fungi into the cambial layer of the tree^[11]^, potentially affecting initial decomposition of the wood as well as adding significant N to the phloem^[12]^. SPB larvae appear to feed mostly on these beneficial fungi, and less on the actual wood itself.

This wood invasion by a defined community of fungi and invertebrates likely shifts log decomposition down a different trajectory than the decomposition of a tree that was killed by lightning, chain saw, drought, etc. The fungus *Ophiostoma minus* Syd. & P. Syd. is imported by the beetle in varying quantities. This fungus grows rapidly and into the sapwood. The two mycangial fungi, of which beetles always carry one, are *Entomocorticium* sp. A and *Ceratocystiopsis ranaculosus* T. J. Perry & J. R. Bridges. Their relative abundance is quite variable from place to place and time to time^[13, 14]^. Both are very slow growing and apparently do not digest xylem much or at all. This beetle-fungal complex is additionally joined by a bacterium that produces antibiotics that help protect *Entomocorticium* from being overgrown by *O. minus*^[15]^. *Entomocorticium* abundance might influence *O. minus* abundance, which might influence the decomposition trajectory of the wood and the fungal communities that are involved. Different outcomes of competition between these fungi depend on the timing and sequence of inoculation by these competing fungal species^[11]^. Changes in the Norway spruce (*Picea abies*) wood-inhabiting fungal community showing signs of bark beetle infection differ from unaffected wood or wood with simulated beetle sized holes^[16]^. This suggests that beetles are likely to alter the trajectory of fungal succession compared to unaffected wood. The nature of the community of early fungal colonizers of wood significantly influences the fungal species richness and rates of wood decomposition^[17]^. Given that fungi compete with each other by chemical means, it is possible that prior colonization of wood by these introduced fungi will compete against the native wood rotting fungal species that will decompose the coarse woody material, allowing longer residence time of this fuel on the forest floor. Although bark volume represents about 12%−13% of a bole^[18]^, this represents the first point of combustible fuel of a fallen tree. Hence, we believe that an understanding of its rate of decomposition and the fungal community responsible for it was pertinent to this study.

Analysis of fungal communities is now routinely performed using molecular tools^[19]^. Similar to the study of Leonhart et al.^[20]^, we used molecular methods (PCR and Illumina metabarcoding approaches) to identify the fungal community of coarse woody material during initial phases of wood decomposition that were, or were not infested, by bark beetles. These methods identified more observational taxonomic units (OTUs) than other methods^[21]^. Other studies measuring succession of fungi in wood decay have used 454 pyrosequencing^[22−24]^ or denatured gradient gel electrophoresis (DGGE)^[25]^. The decomposition rate of this material (mass loss) was related to the changes in fungal community composition over a 42-month period. The colonization of wood by decomposer organisms is strongly influenced by which organisms first colonize the resource^[17, 26]^, wood quality, edaphic conditions and degree of contact with soil^[27]^.

Our hypothesis was that initial colonization by fungi specific to bark beetles will alter the successional pattern of colonization by wood decomposing fungi (as identified by Ottosson et al.^[24]^) and limit the rate of decomposition of the woody material. We also chose to look at both wood and bark decomposition as bark has higher contents of polyphenols than wood (up to three times)^[28, 29]^ and is, thus, likely to contain a different fungal community than wood.

## RESULTS

### Wood mass loss

**Figure 1:**
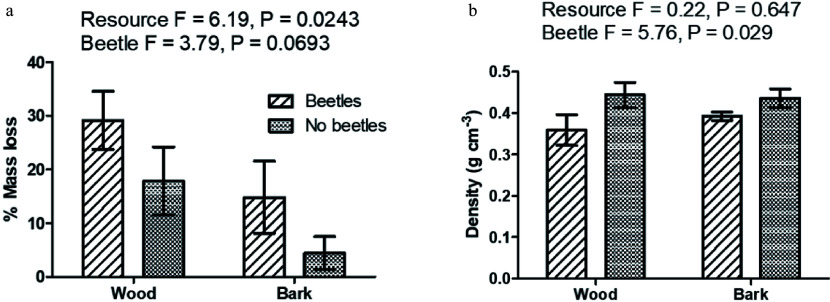
Percent mass loss (a) and density (b) of wood and bark at final (42 month) harvest. Bars are standard error of the mean. Statistics are from a two-way ANOVA, degrees of freedom 1,16 for both resource and beetle. The interaction between resource and beetle was not significant (F_(1,16)_ = 0.01, *P* = 0.9361).

Mass loss of wood and bark over time showed an overall pattern similar to that seen at the end of the field incubation (42 months). The lack of significant differences in a time series analysis is attributed to the errors introduced in the calculation of dry mass. Total dry mass of the wood or bark sample was corrected for the amount of sampled removed for molecular analysis. As this removed material had to be fresh, the calculation of its dry weight equivalent may not have been accurate. Percentage mass loss and resource bulk density for wood and bark are shown in Fig. 1.

From a time series ANOVA, there was a significant difference in wood mass loss over time (F_(3,32)_ = 4.35, *P* = 0.0111), but not for beetle infestation status (F_(3,32)_ =0.83, *P* = 0.4881). For bark, neither factor was significant (time, F_(3,32)_ = 0.19, *P* = 0.9057; beetle infestation status, F_(3,32)_ = 0.02, *P* = 0.9025).

Both resource types (wood and bark) decomposed faster when they had been previously infested by southern pine bark beetles (significant at α = 0.1). Bark decomposed at a significantly slower rate than wood (*P* = 0.024). Wood and bark density showed an expected inverse relationship of mass loss with density. Density of beetle infested resources were significantly lower than for non-beetle infested resource (*P* = 0.029), but there was no significant difference in density between bark and wood (*P* = 0.647).

### Molecular analysis of fungal community

Non-fungi reads were eliminated from further analyses, and only matches to the Fungi branch in the MEGAN tree output were used. As there were multiple sequence reads for each fungal OTU the total number of reads was summed over these multiple reads.

**Figure 2:**
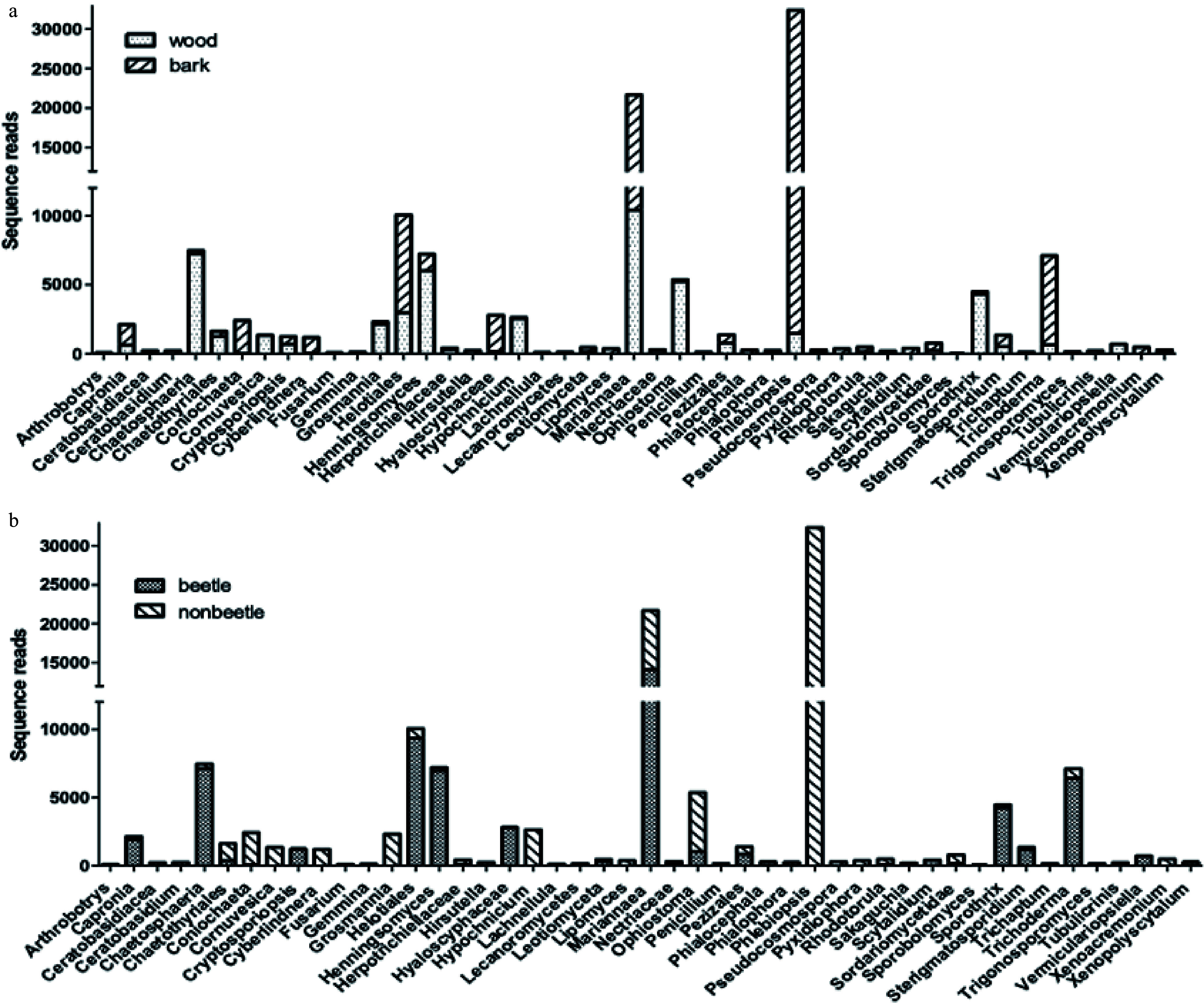
Sequence reads of fungal taxa showing significant differences (ANOVA at *P* < 0.01) between resource types (a) and between SPB infested and uninfested resources (b) in samples prior to incubation in litter bags in the field.

At the time of collection, there were differences in the fungal communities of wood and bark and between wood affected by beetles and that not affected (Fig. 2). This figure shows the fungal species that were statistically different (*P* < 0.01) between resource type and beetle infection. Of the dominant fungal taxa (greatest number of reads), *Phlebiopsis* is more abundant in bark than wood and almost exclusively related to non-beetle wood. Helotiales and *Trichoderma* are likewise more abundant in bark, but associated with beetle infested resource. In comparison, *Chaetosphaeria* and *Mariannaea* are more prominent in wood than bark and highly associated with beetle infestation.

**Figure 3:**
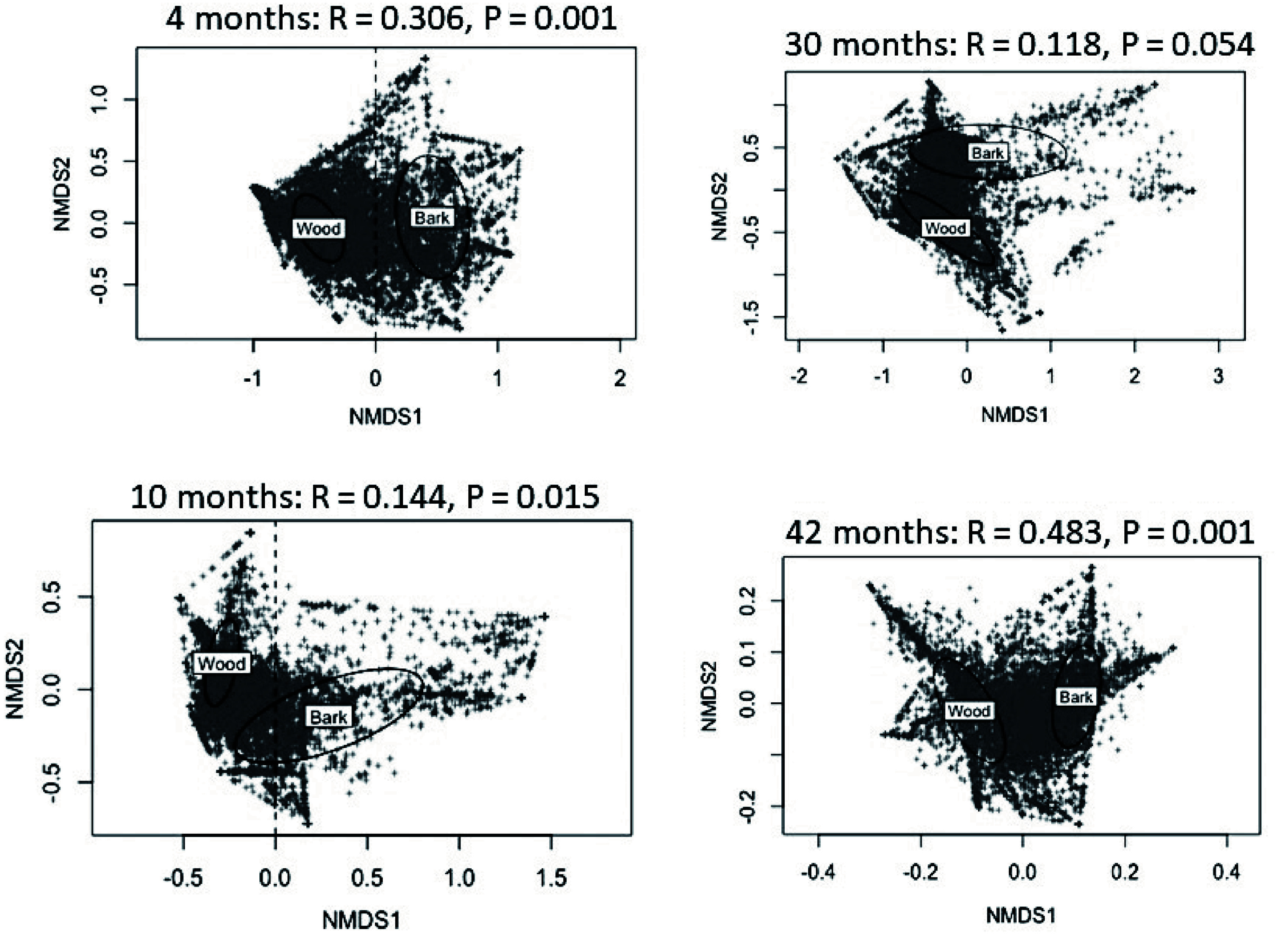
Differences in fungal community composition between wood and bark as visualized in a non-metric multidimensional scaling (NMDS) analysis for each sampling time. There were no significant differences in communities between beetle infested and uninfested resources.

Results of NMDS analysis of the OTU data, over incubation time, is shown in Fig. 3. There was a significant difference in the fungal community between wood and bark at each of the sampling times. A difference between beetle infested and uninfested resource was only found at the 4 month harvest (*P* = 0.038) and not at subsequent harvest dates.

**Figure 4:**
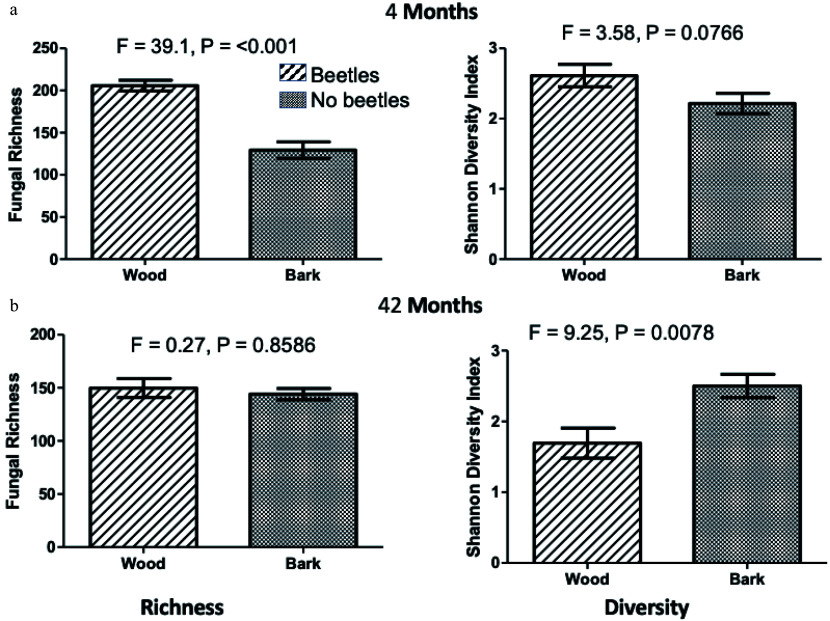
Fungal OTU community richness and diversity in wood and bark at 4 (a) and 42 (b) months of field incubation. Bars are standard errors of means. There were no significant differences between resources at intermediate harvest times. Analysis by two-way ANOVA with degrees of freedom as 1,16.

There was no significant difference in richness or diversity between beetle and no-beetle resource, but there were significant differences between these metrics for wood and bark at 4 months and diversity at 42 months (Fig. 4). This shifted to a higher diversity in wood than bark at 4 months to a lower diversity in wood at 42 months.

**Figure 5:**
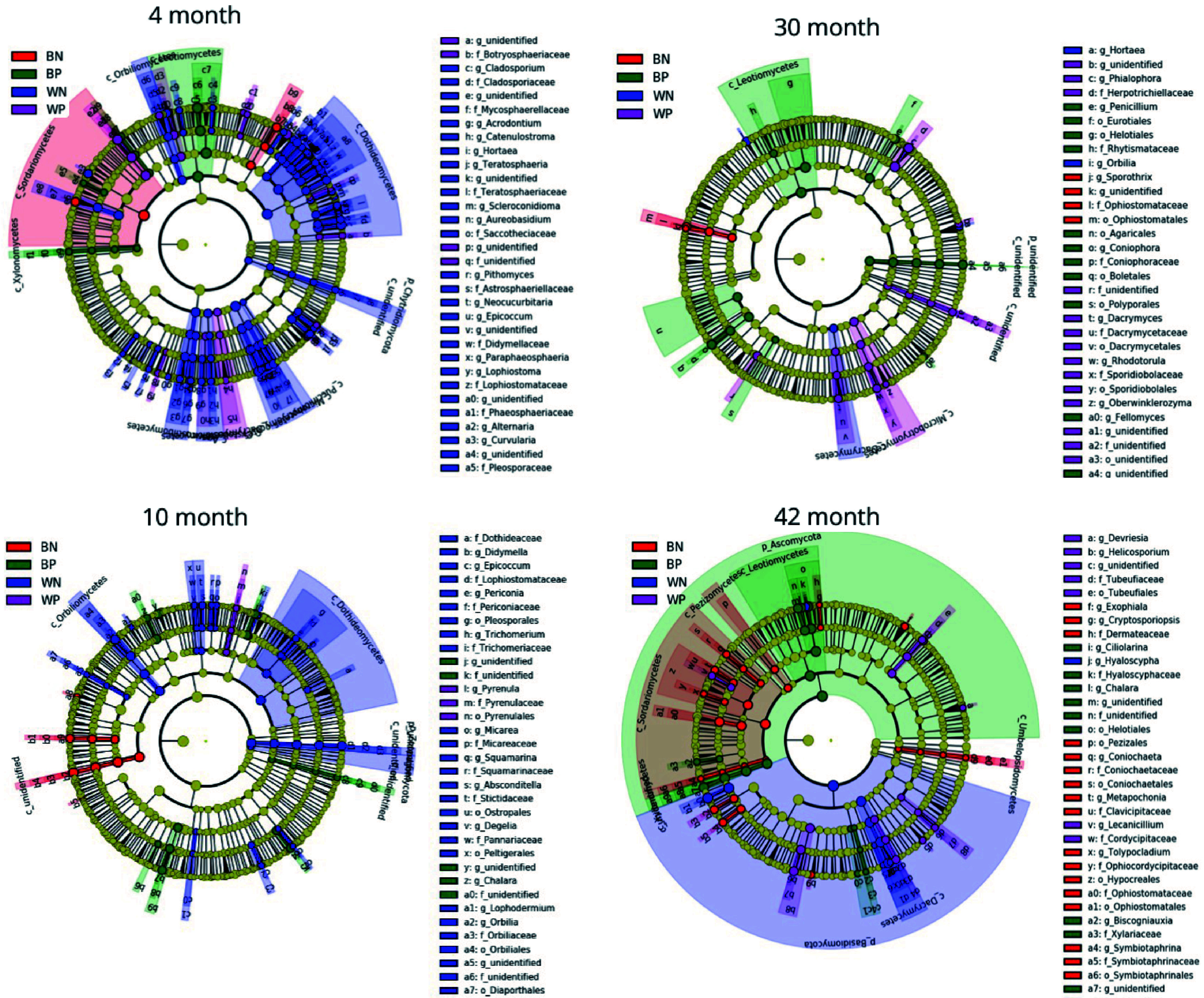
Cladograms of fungal taxa (highlighted by small circles and by shading) showing differences in abundance values (according to LEfSe) in the four classes BN (uninfested bark), BB (beetle infested bark), WN (uninfested wood) and WB (beetle infested wood) for each of the sampling times.

By analyzing each OTU between beetle colonization and between resource type we could see which OTU was significantly more abundant in wood than bark (or the converse) or higher abundance in beetle infested than non-beetle infested resource (or the converse) at each harvest time (Table 1). Wood supported a higher number of fungal OTUs than bark, but this number declined over incubation time. Bark specific fungal OTUs were low at 4 months but then stabilized at double the number for the duration of the incubation. Specificity of fungal OTUs to beetle colonized resource was low and high numbers of OTUs were found at 4 and 30 months in uninfested wood. The actual OTU classification between resource type are given in Supplemental Table S1 and between beetle and uninfested resource in Supplemental Table S2. Cladograms of fungal taxa showing those taxa that are significantly more abundant in each of the resource types over time are shown in Fig. 5. At the 4 month harvest, Sordariomycetes were represented in all resource types, whereas Dothideomycetes were only represented in wood. At 10 months, Leotiomycetes were represented in beetle infested bark, but Ophiostomatales in uninfested bark. The ratio of ascomycete reads to basidiomycete reads was higher in the initial stage of decomposition than later stages and the ratio was much higher in bark than in wood (Table 2), where Ascomycota appear to be dominant in bark and Basidiomycota in wood (Fig. 5).

**Table 1:** Number of fungal OTU taxa with significantly higher (α = 0.1) total sequence reads between resource types and between beetle and non-beetle resources.

	Wood	Bark	Beetle	Non-beetle
4 month	80	6	8	50
10 month	23	14	5	6
30 month	17	14	3	26
42 month	15	12	3	5

**Table 2:** Ratio of total ascomycete to basidiomycete reads between resources over time. There was no significant difference in this ratio between beetle infested and uninfested resources. Superscripts within columns indicate significant differences between means with Time (F = 3.37, *P* = 0.0239) and Resource (F = 5.19, *P* = 0.0261).

	Asco:Basidio ratio				Asco:Basidio ratio	
Time (mo)	Mean	SEM		Resource	Mean	SEM
4	156.02 ^A^	61.90		Bark	113.9 ^A^	24.65
10	2.33 ^B^	0.764		Wood	35.81 ^B^	5.40
30	71.35 ^AB^	14.98				
42	69.83 ^AB^	29.48				

Within bark, there is very little continuity of bark specific fungi over time. Only *Tolypocladium*, *Penicillium* and Heliotiales occur twice as more bark specific fungi. Fungal richness in bark increases after the first harvest time and then remains constant over time, whereas the richness of wood related fungi declines continually over time. In wood, *Piskurozyma* occurs at a significantly higher rate over all sampling times; *Oberwinklerozyma*, *Dacromyces*, *Lophodermium* and *Cladosporium* occur significantly higher in wood in three out of four harvest times. *Rhodotorula*, *Slooffia*, *Teratospharia*, *Trichomerium* and Chytridomycota occur mainly in early stages of wood decay.

Comparison of beetle infested, or uninfested resources (wood and bark combined) shows much less specificity of fungal groups. Only *Phialophora* occurs more than once in beetle infested wood, while *Ophiostoma*, *Cladosporium* and *Sporobolomyces* occur in two time periods in uninfested resources. It is interesting that significantly higher occurrence of *Ophiostoma* (2 time points) and *Sporothrix* (second harvest) occur in non-beetle resources as these are two fungal genera that are closely associated with bark beetles. Fungal richness in both beetle and non-beetle resources shows a trend of decline over time.

## DISCUSSION

Although it is appreciated that small pieces of wood and bark are likely not to decay at the same rate as whole logs, we have shown that there is a strong likelihood that these resources decompose more rapidly after beetle attack than uninfested resources. Hence, beetle attacked wood poses no greater fire risk than other dead coarse woody debris, due to a different rate of decomposition.

Interactions between fungi can significantly influence physiological function, such as pathogenicity and decomposition^[30]^. For example, *Trichoderma* is antagonistic to *Phellinus noxius* and significantly decrease wood decomposition rate^[31]^. The introduction of fungi carried by bark beetles is an inoculation by a number of taxa that are useful for beetles by their ability to concentrate nitrogen to feed their larval stages^[12]^. However, these beetles may import many more fungal taxa of which many are wood decomposers. Some 21 fungal taxa were found in the gallery region of beetles infesting Norway spruce, of which 12 were ascomycetes and nine basidiomycetes^[31]^. Ambrosia beetles infesting pines introduced 46 fungal OTUs of which 35% were yeast and 19% Ophiostomatean^[32]^. These introductions facilitate establishment of wood decay fungi^[16]^. Thus, it is not surprising that the recently beetle-attacked wood that we used in our study contained a high fungal diversity (Fig. 2), even before deployment for our decomposition experiment, and that the fungal community differed between beetle infested and uninfested resource^[16]^.

Our studies showed significant difference in fungal communities developing in wood and bark, but much less between beetle infested and uninfested resources (wood or bark). We did not see a clear relationship between fungal richness and resource mass loss^[17]^ but fungal diversity in bark at 42 months was much higher than in wood, suggesting that possible competitive interactions within this highly diverse community were reducing decomposition rate, in addition to the presence of defense chemicals.

We chose to measure decomposition rates and fungal communities in both wood and bark tissue as there is less information in the literature regarding the decomposition of bark^[33]^. Bark tends to have higher levels of defense compounds than wood, with *Pinus pinea* having 37.5% lignin and polyphenol content and 37% polysaccharide content, compared to wood^[29]^. Similarly, in *Eucalyptus globulus*, total phenol content is 31.4 mg g^−1^ compared to wood concentration of 9.4 mg g^−1^^[28]^. By sampling bark from logs over a 41 year period^[23]^ revealed that fungal richness increased to a plateau and then declined over that time period as bark mass decreased. The fungal successional changes were for ascomycete domination in early decay stages to basidiomycete and ectomycorrhizal dominated communities at later stages of decay. Our study covered a very early stage of decomposition but we could see a higher ratio of ascomycetes to basidiomycetes, which was reduced over time (Table 2), similar to that of Kazartzev et al.^[23]^. Interestingly, ascomycetes appear to dominate over basidiomycetes in bark to much greater extent than in wood (Table 2), suggesting a different fungal community effecting decomposition of the two resources. In contrast to these results, Kubatova et al.^[22]^ found a dominance of basidiomycetes in areas of the same Norway spruce log that had been colonized or not by bark beetles.

From ANOVAS of abundance (sequence reads) of individual fungi within the fungal community of both wood and bark, we can see trends in differential successional patterns between resources impacted by beetles, or not (Supplemental Table S1 and S2). Certainly, we see distinct differences in fungal communities between wood and bark. Our study was of short duration, so it would be informative to look at this system over a longer time period. It may then be possible to link comparative decomposition rates with specific fungal taxa or groups of taxa and then link that to fungal physiological functions.

## MATERIALS AND METHODS

### Selection of wood for decomposition

Wood was collected from pitch pine (*Pinus rigida*) trees that had been felled following a bark beetle attack. Wood was taken from a site north of Viking Yachts on Rt 9 at the eastern edge of the New Jersey pine barrens in August 2015. Two adjacent trees were used, one with indications of beetle infestation and the other (felled as part of the felling protocol) which showed minimal infestation. Beetle activity within the wood was determined by the density of beetle emergence holes (beetle wood with an average of 11.2 holes per 25 cm^−2^ area vs non-beetle at 1.2 holes cm^−2^ (F_1,14_ = 78.4, *P* < 0.0001)). On return to the laboratory, beetle and non-beetle wood and bark for incubation was prepared similarly. Bark was separated from the wood and wood samples taken from the first cm under the bark. Pieces of wood and bark approximately 5 × 1 × 1 cm (size was variable depending on friability of bark and ability to cut the wood) using a woodworker's chisel. All samples were taken from the same small (20 cm long) internodal trunk sample as it has been shown that there is significant fine-scale differences in fungal communities in wood^[22]^ and physicochemical differences between nodal and internodal wood^[27]^.

### Experimental design

The density of sub samples of wood and bark were determined by displacement of water, then dried at 70 °C to determine dry mass equivalent of air dried material. One piece of wood and one of bark were placed in litter bags (1 mm mesh size constructed at 5 × 5 cm from fiberglass fly screen). Five replicate bags containing both wood and bark (with or without beetle infestation) were placed on the soil surface under a pine canopy at the Rutgers Pinelands Field Station in a randomized block design, for each of four sampling times (a total of 80 litter bags). Bags were deployed on August 20 2015 on the surface of about a 2 cm litter and humus horizon over the sandy soil of the pine barrens, which typically has a pH of about 4.5.

Bags were harvested at 4 months (Dec 4 2015), 10 months (June 9 2016), 30 months (Feb 15 2017) and 42 months (Feb 18 2018). The density of each wood and bark sample was determined by displacement, blotted dry with paper towel and a known mass of sample taken by drilling with a 1/8" drill for molecular analysis. The drill bit was alcohol flamed between samples^[22, 25, 26]^. The remaining sample was dried at 70 °C to determine mass loss (with correction of sub sample removed for molecular analysis). Wood and bark loss data were statistically analyzed by two way ANOVA after checking for homogeneity of variance.

### Fungal molecular analysis

Approximately 0.1 g of wood or bark was lysed and total DNA extracted using the DNeasy PowerSoil Isolation kit (Qiagen, Maryland). PCR amplification of the ITS region^[16, 23, 25]^ provided metabarcoding using three annealing temperatures of 50, 55 and 58 °C over 30 cycles. Next-gen sequencing was performed on 5 µl of three pooled PCR products per sample using an Illumina Miseq.

Removal of sequencing adapters, PCR primers and low-quality bases, as well as the merging of forward and reverse reads was performed through the CLC Genomics Workbench v8.5.1. Quality control parameters were set to reject any sequences < 100 bp long. Sequences were de-replicated using the "fastx_uniques" command in USEARCH 9.0^[34]^. Sequences were sorted by size and singleton sequences (those with abundance of < 2) were discarded from further analysis using the "sortbysize" in USEARCH 9.0^[34]^. Singletons were removed because they were likely artifacts of the amplification process^[35]^.

The USEARCH 9.0^[34]^ algorithm was used to cluster and combine sequence files for downstream statistical analysis. Identical reads (length and composition) were compiled based on pairwise similarity of 97% using the "cluster otus" command in USEARCH 9.0^[34]^ and grouped together as Observational Taxonomic Units (OTUs). During clustering, sequences were checked for chimeric sequences. Alignment of OTU sequences to curated databases was achieved by BLAST with GenBank NT and UNITE v.8.0^[36]^ databases. BLASTn results were observed and organized in MEGAN Community Edition, v.6.15^[37]^ with the default lowest common ancestor (LCA) parameters (minimum score of 50.0, minimum support percent of 0.01, and with minimum-complexity filter off). To eliminate any non-fungi from further analyses, only matches to the Fungi branch in the MEGAN tree output were used. The BLASTn outputs were also analyzed with MEGAN for the taxonomic rank of interest. A taxon table was constructed in Rstudio, v 1.2.5033 (2019) from merging the USEARCH clustering output files containing the number of sequences and the OTU names with the MEGAN output file containing the OTU names and the corresponding taxonomic rank. The resulting taxon table was input into the online LEfSe (https://huttenhower.sph.harvard.edu/galaxy) statistical program and analyzed with the default statistical settings and with the multi-class analysis set to the less strict one-against-all option^[38]^.

Community differences between samples were distinguished using non-metric multidimensional scaling (NMDS)^[20]^. Total sequence reads for each of the major fungal groups identified from fungal OTUs were analyzed by analysis of variance (ANOVA) between the resources wood and bark (irrespective of beetle infestation) and for both resource types between beetle infested and uninfested (irrespective of resource type)^[20]^. Significant differences in sequence abundance are reported at α = 0.1.

## SUPPLEMENTARY DATA

Supplementary data to this article can be found online.
