# Supplementary material for: Influence of southern pine beetle on fungal communities of wood and bark decomposition of coarse woody debris in the New Jersey pine barrens
Source: For Res (Fayettev). 2021 Oct 25;1:17. doi: 10.48130/FR-2021-0017 (PMC11524313; doi:10.48130/FR-2021-0017)
Supplement: Supplementary file 1 — Supplementary data to this article can be found online. [file FR-2021-0017-S1.zip › 10.48130_FR-2021-0017-Suppl-TableS2.docx]

| **Table 2. Total sequence read abundance delineating fungal OTUs significantly higher in beetle infested (B) or uninfested (N) resources (wood and bark) over time. Differences are significant at α= 0.1.** | | | | | | | | | | | |
| --- | --- | --- | --- | --- | --- | --- | --- | --- | --- | --- | --- |
|  |  |  |  |  |  |  |  |  |  |  |  |
| **Time 1** | | | **Time 2** | | | **Time 3** | | | **Time 4** | | |
| Capronia villosa | B |  | Leotiomyceta | B |  | Candelariales | B |  | Leptosphaeria | B |  |
| Ciliolarina | B |  | Orbiliaceae | B |  | Hyaloscyphaceae | B |  | Nigrospora | B |  |
| Drechslerella | B |  | Phialophora | B |  | Microsporomyces | B | 3 | Phanerochaete | B | 3 |
| Hirsutella thompsonii | B |  | Phialophora sp. H30 | B |  |  |  |  |  |  |  |
| Massarineae | B |  | Scytalidium | B | 5 | Absconditella | N |  | Chaetothyriales | N |  |
| Phialophora | B |  |  |  |  | Agaricostilbomycetes | N |  | Coniochaeta | N |  |
| Sordariales | B |  | Chaetomium | N |  | Basidiobolus | N |  | Devriesia | N |  |
| Tolypocladium | B | 8 | Lecanoraceae | N |  | Boletales | N |  | Pertusariales | N |  |
|  |  |  | Lecophagus | N |  | Capnodiales | N |  | Symbiotaphrina | N | 5 |
| Antrodiella | N |  | Mariannaea elegans | N |  | Ceratobasidiaceae | N |  |  |  |  |
| Ceramothyrium carniolicum | N |  | Ophiostoma angusticollis | N |  | Chrysozymaceae | N |  |  |  |  |
| Chionosphaera cuniculicola | N |  | Sporothrix | N | 6 | Cladosporium | N |  |  |  |  |
| Chrysozymaceae | N |  |  |  |  | Cuniculitremaceae | N |  |  |  |  |
| Chytridiomycota | N |  |  |  |  | Degelia | N |  |  |  |  |
| Cladosporium | N |  |  |  |  | Desmazierella acicola | N |  |  |  |  |
| Claussenomyces | N |  |  |  |  | Devriesia | N |  |  |  |  |
| Cordycipitaceae | N |  |  |  |  | Dothideomycetes | N |  |  |  |  |
| Cortinarius | N |  |  |  |  | Erythrobasidiales | N |  |  |  |  |
| Cystobasidiomycetes | N |  |  |  |  | Gaeumannomyces | N |  |  |  |  |
| Dothideomycetes | N |  |  |  |  | Kurtzmanomyces | N |  |  |  |  |
| Dothioraceae | N |  |  |  |  | Lopadostoma | N |  |  |  |  |
| Epicoccum | N |  |  |  |  | Meliniomyces | N |  |  |  |  |
| Exobasidium | N |  |  |  |  | Monochaetia | N |  |  |  |  |
| Fellozyma inositophila | N |  |  |  |  | Nectriaceae | N |  |  |  |  |
| Fomitopsidaceae | N |  |  |  |  | Orbilia | N |  |  |  |  |
| Fonsecazyma | N |  |  |  |  | Parmeliaceae | N |  |  |  |  |
| Fusarium | N |  |  |  |  | Phaeomoniella | N |  |  |  |  |
| Hortaea | N |  |  |  |  | Pleosporomycetidae | N |  |  |  |  |
| Lecanoromycetes | N |  |  |  |  | Radulomyces | N |  |  |  |  |
| Microbotryomycetes | N |  |  |  |  | Sporobolomyces | N | 26 |  |  |  |
| Microcyclospora | N |  |  |  |  |  |  |  |  |  |  |
| Morenoina calamicola | N |  |  |  |  |  |  |  |  |  |  |
| Nectriaceae | N |  |  |  |  |  |  |  |  |  |  |
| Oberwinklerozyma | N |  |  |  |  |  |  |  |  |  |  |
| Ogataea | N |  |  |  |  |  |  |  |  |  |  |
| Omphalotaceae | N |  |  |  |  |  |  |  |  |  |  |
| Ophiostoma | N |  |  |  |  |  |  |  |  |  |  |
| Phaeosphaeriaceae | N |  |  |  |  |  |  |  |  |  |  |
| Phaeotremella | N |  |  |  |  |  |  |  |  |  |  |
| Phlebiella borealis | N |  |  |  |  |  |  |  |  |  |  |
| Pleosporineae | N |  |  |  |  |  |  |  |  |  |  |
| Pleosporomycetidae | N |  |  |  |  |  |  |  |  |  |  |
| Pseudobensingtonia | N |  |  |  |  |  |  |  |  |  |  |
| Pseudotremella allantoinivorans | N |  |  |  |  |  |  |  |  |  |  |
| Saccharomycetales | N |  |  |  |  |  |  |  |  |  |  |
| Septobasidium | N |  |  |  |  |  |  |  |  |  |  |
| Sirotrema translucens | N |  |  |  |  |  |  |  |  |  |  |
| Slooffia | N |  |  |  |  |  |  |  |  |  |  |
| Sporobolomyces | N |  |  |  |  |  |  |  |  |  |  |
| Sydowia polyspora | N |  |  |  |  |  |  |  |  |  |  |
| Symbiotaphrina | N |  |  |  |  |  |  |  |  |  |  |
| Teratosphaeria | N |  |  |  |  |  |  |  |  |  |  |
| Tremella | N |  |  |  |  |  |  |  |  |  |  |
| Trichomerium | N |  |  |  |  |  |  |  |  |  |  |
| Trimorphomycetaceae | N |  |  |  |  |  |  |  |  |  |  |
| Ustilaginaceae | N |  |  |  |  |  |  |  |  |  |  |
| Valsaceae | N |  |  |  |  |  |  |  |  |  |  |
| Vishniacozyma | N | 50 |  |  |  |  |  |  |  |  |  |
