# Supplementary material for: Influence of southern pine beetle on fungal communities of wood and bark decomposition of coarse woody debris in the New Jersey pine barrens
Source: For Res (Fayettev). 2021 Oct 25;1:17. doi: 10.48130/FR-2021-0017 (PMC11524313; doi:10.48130/FR-2021-0017)
Supplement: Supplementary file 1 — Supplementary data to this article can be found online. [file FR-2021-0017-S1.zip › 10.48130_FR-2021-0017-Suppl-TableS1.docx]

| **Table 1. Total sequence read abundance delineating fungal OTUs significantly higher in bark (B) or wood (W) over time. Differences are significant at α= 0.1.** | | | | | | | | | |  |  |
| --- | --- | --- | --- | --- | --- | --- | --- | --- | --- | --- | --- |
|  |  |  |  |  |  |  |  |  |  |  |  |
| **Time 1** | | | **Time 2** | | | **Time 3** | | | **Time 4** | | |
| Helotiales | B |  | Ballistosporomyces taupoensis | B |  | Athelia | B |  | Chalara | B |  |
| Lecanicillium | B |  | Fellomyes | B |  | Chytridiomycota | B |  | Coniochaeta | B |  |
| Malassezia | B |  | Fusarium | B |  | Erythrobasidiales | B |  | Cryptosporiopsis | B |  |
| Ophiocordycipitaceae | B |  | Hyaloscyphaceae | B |  | Gaeumannomyces | B |  | Gelasinospora | B |  |
| Penicillium | B |  | Inocybe | B |  | Kazachstania | B |  | Helotiales | B |  |
| Tolypocladium | B | 6 | Ostropomycetidae | B |  | Mariannaea | B |  | Lactarius | B |  |
|  |  |  | Phaeomoniella | B |  | Microbotryomycetes | B |  | Metapochonia bulbillosa | B |  |
| Absconditella | W |  | Pleosporineae | B |  | Mucor | B |  | Mycena | B |  |
| Acrodontium | W |  | Propolis farinosa | B |  | Mycena | B |  | Septobasidium | B |  |
| Agaricostilbomycetes | W |  | Pyrenochaeta | B |  | Penicillium | B |  | Sporocadaceae | B |  |
| Antrodiella | W |  | Rhytismatales | B |  | Pyrenochaeta | B |  | Symbiotaphrina | B |  |
| Aureobasidium | W |  | Sporothrix | B |  | Sporidiobolales | B |  | Ustilaginomycotina | B | 12 |
| Beauveria | W |  | Tolypocladium | B |  | Sporothrix | B |  |  |  |  |
| Boletales | W |  | Trichosporonaceae | B | 14 | Symbiotaphrina | B | 14 | Acarosporaceae | W |  |
| Botryosphaeriales | W |  |  |  |  |  |  |  | Cerinomyces | W |  |
| Bullera | W |  | Acremonium | W |  | Agaricostilbomycetes | W |  | Cladophialophora | W |  |
| Bulleribasidiaceae | W |  | Agaricaceae | W |  | Cladosporium | W |  | Cladosporium | W |  |
| Capnobotryella renispora | W |  | Chrysozymaceae | W |  | Cystobasidiomycetes | W |  | Dacrymycetes | W |  |
| Capnodiales | W |  | Chytridiomycota | W |  | Devriesia | W |  | Devriesia | W |  |
| Capronia | W |  | Dacrymyces | W |  | Lophodermium | W |  | Geastrales | W |  |
| Ceramothyrium carniolicum | W |  | Devriesia | W |  | Micarea | W |  | Helicosporium | W |  |
| Cerinomyces | W |  | Didymosphaeriaceae | W |  | Oberwinklerozyma | W |  | Hortaea | W |  |
| Chionosphaera cuniculicola | W |  | Hannaella | W |  | Orbilia | W |  | Lophiostomataceae | W |  |
| Chrysozymaceae | W |  | Kockovaella | W |  | Phaeosphaeriaceae | W |  | Lophodermium | W |  |
| Chytridiomycota | W |  | Microsporomyces | W |  | Phaeotremella | W |  | Micarea | W |  |
| Cladosporium | W |  | Neocucurbitaria | W |  | Piskurozyma | W |  | Piskurozyma | W |  |
| Colacogloea | W |  | Oberwinklerozyma | W |  | Pleosporomycetidae | W |  | Rhinocladiella | W |  |
| Coniophora | W |  | Oberwinklerozyma yarrowii | W |  | Pyrenula | W |  | Tubeufiaceae | W | 15 |
| Coniothyrium | W |  | Ochrocladosporium | W |  | Radulomyces | W |  |  |  |  |
| Cyberlindnera | W |  | Phialophora | W |  | Rhizosphaera | W |  |  |  |  |
| Cystobasidiomycetes | W |  | Phialophora sp. H30 | W |  | Squamarina gypsacea | W |  |  |  |  |
| Dacrymycetes | W |  | Piskurozyma | W |  | Trichomerium | W | 17 |  |  |  |
| Dacryobolaceae | W |  | Rhodotorula | W |  |  |  |  |  |  |  |
| Diaporthe | W |  | Slooffia | W |  |  |  |  |  |  |  |
| Didymellaceae | W |  | Teratosphaeriaceae | W |  |  |  |  |  |  |  |
| Dothideomycetes | W |  | Tulasnellaceae | W |  |  |  |  |  |  |  |
| Epicoccum | W |  | Venturiales | W |  |  |  |  |  |  |  |
| Erythrobasidium | W |  | Xylariales | W | 23 |  |  |  |  |  |  |
| Exobasidium | W |  |  |  |  |  |  |  |  |  |  |
| Fomitopsidaceae | W |  |  |  |  |  |  |  |  |  |  |
| Fonsecazyma | W |  |  |  |  |  |  |  |  |  |  |
| Fusarium | W |  |  |  |  |  |  |  |  |  |  |
| Hortaea | W |  |  |  |  |  |  |  |  |  |  |
| Hyphoderma | W |  |  |  |  |  |  |  |  |  |  |
| Kwoniella | W |  |  |  |  |  |  |  |  |  |  |
| Lecanorales | W |  |  |  |  |  |  |  |  |  |  |
| Lophiostoma | W |  |  |  |  |  |  |  |  |  |  |
| Lophodermium | W |  |  |  |  |  |  |  |  |  |  |
| Massarineae | W |  |  |  |  |  |  |  |  |  |  |
| Microbotryomycetes | W |  |  |  |  |  |  |  |  |  |  |
| Morenoina calamicola | W |  |  |  |  |  |  |  |  |  |  |
| Mycosphaerellaceae | W |  |  |  |  |  |  |  |  |  |  |
| Neocucurbitaria | W |  |  |  |  |  |  |  |  |  |  |
| Neopseudocercosporella capsellae | W |  |  |  |  |  |  |  |  |  |  |
| Oberwinklerozyma | W |  |  |  |  |  |  |  |  |  |  |
| Ochrocladosporium | W |  |  |  |  |  |  |  |  |  |  |
| Orbilia | W |  |  |  |  |  |  |  |  |  |  |
| Papiliotrema | W |  |  |  |  |  |  |  |  |  |  |
| Phaeosphaeriaceae | W |  |  |  |  |  |  |  |  |  |  |
| Phaeotremella | W |  |  |  |  |  |  |  |  |  |  |
| Phialophora | W |  |  |  |  |  |  |  |  |  |  |
| Phlebia | W |  |  |  |  |  |  |  |  |  |  |
| Phlebiella borealis | W |  |  |  |  |  |  |  |  |  |  |
| Piskurozyma | W |  |  |  |  |  |  |  |  |  |  |
| Pleosporales | W |  |  |  |  |  |  |  |  |  |  |
| Proliferodiscus earoleucus | W |  |  |  |  |  |  |  |  |  |  |
| Psathyrella | W |  |  |  |  |  |  |  |  |  |  |
| Pseudobensingtonia | W |  |  |  |  |  |  |  |  |  |  |
| Pseudotremella allantoinivorans | W |  |  |  |  |  |  |  |  |  |  |
| Rhodotorula | W |  |  |  |  |  |  |  |  |  |  |
| Saccharomycetales | W |  |  |  |  |  |  |  |  |  |  |
| Septobasidium | W |  |  |  |  |  |  |  |  |  |  |
| Slooffia | W |  |  |  |  |  |  |  |  |  |  |
| Sordariales | W |  |  |  |  |  |  |  |  |  |  |
| Steccherinaceae | W |  |  |  |  |  |  |  |  |  |  |
| Stereum | W |  |  |  |  |  |  |  |  |  |  |
| Sydowia polyspora | W |  |  |  |  |  |  |  |  |  |  |
| Sympoventuriaceae | W |  |  |  |  |  |  |  |  |  |  |
| Teratosphaeria | W |  |  |  |  |  |  |  |  |  |  |
| Thelephoraceae | W |  |  |  |  |  |  |  |  |  |  |
| Trechispora | W |  |  |  |  |  |  |  |  |  |  |
| Trichomerium | W |  |  |  |  |  |  |  |  |  |  |
| Trimorphomycetaceae | W |  |  |  |  |  |  |  |  |  |  |
| Valsaceae | W |  |  |  |  |  |  |  |  |  |  |
| Vonarxula javanica | W |  |  |  |  |  |  |  |  |  |  |
| Xenasmatella | W |  |  |  |  |  |  |  |  |  |  |
| Xenospadicoidaceae | W | 80 |  |  |  |  |  |  |  |  |  |
